# A Treatise on the Horse, and His Diseases

**Published:** 1879-07

**Authors:** 


A TREATISE ON THE HORSE, AND HIS DISEASES. By B. J. Kindall M.D., Enosburgh Fall, Vt. Claremont Manufacturing Company publishers, N. H., 1879.

A book in pamphlet form containing a description of horse diseases with their treatment. Facts are presented and treated of in a practical and substantial manner, rendering the book very desirable to those who may engage in veterniary practice.

